# *t*-Bu_2_SiF-Derivatized D_2_-Receptor Ligands: The First SiFA-Containing Small Molecule Radiotracers for Target-Specific PET-Imaging

**DOI:** 10.3390/molecules16097458

**Published:** 2011-09-02

**Authors:** Ljuba Iovkova-Berends, Carmen Wängler, Thomas Zöller, Georg Höfner, Klaus Theodor Wanner, Christian Rensch, Peter Bartenstein, Alexey Kostikov, Ralf Schirrmacher, Klaus Jurkschat, Björn Wängler

**Affiliations:** 1 Department of Inorganic Chemistry II, Faculty of Chemistry, TU Dortmund, Otto-Hahn-Str. 6, 44221 Dortmund, Germany; 2 Department of Nuclear Medicine, Ludwig-Maximilians-University, Marchioninistr. 15, 81377 Munich, Germany; 3 Department of Pharmacy, Center for Drug Research, Ludwig-Maximilians-University, Butenandtstr. 7, 81377 Munich, Germany; 4 GE Global Research, 85748 Garching, Germany; 5 McConnell Brain Imaging Centre, Montreal Neurological Institute, McGill University, 3801 University St., Montreal H3A 2B4, QC, Canada

**Keywords:** fluorine, isotopic labeling, positron emission tomography, radiopharmaceuticals, silicon

## Abstract

The synthesis, radiolabeling and *in vitro* evaluation of new silicon-fluoride acceptor (SiFA) derivatized D_2_-receptor ligands is reported. The SiFA-technology simplifies the introduction of fluorine-18 into target specific biomolecules for Positron-Emission-Tomography (PET). However, one of the remaining challenges, especially for small molecules such as receptor-ligands, is the bulkiness of the SiFA-moiety. We therefore synthesized four Fallypride SiFA-conjugates derivatized either directly at the benzoic acid ring system (SiFA-DMFP, SiFA-FP, SiFA-DDMFP) or at the butyl-side chain (SiFA-M-FP) and tested their receptor affinities. We found D_2_-receptor affinities for all compounds in the nanomolar range (*K*_i(SiFA-DMFP)_ = 13.6 nM, *K*_i(SiFA-FP)_ = 33.0 nM, *K*_i(SiFA-DDMFP)_ = 62.7 nM and *K*_i(SiFA-M-FP)_ = 4.21 nM). The radiofluorination showed highest yields when 10 nmol of the precursors were reacted with [^18^F]fluoride/TBAHCO_3_ in acetonitrile. After a reversed phased cartridge purification the desired products could be isolated as an injectable solution after only 10 min synthesis time with radiochemical yields (RCY) of more than 40% in the case of SiFA-DMFP resulting in specific activities >41 GBq/µmol (>1,100 Ci/mmol). Furthermore, the radiolabeled products were shown to be stable in the injectable solutions, as well as in human plasma, for at least 90 min.

## 1. Introduction

Positron-Emission-Tomography (PET) is a non-invasive imaging technique using contrast-agents (radiotracers) labeled with radionuclides such as fluorine-18 which undergo positron emission decay. The resulting positron annihilates with an electron, producing two gamma photons, emitted at a 180° angle, which can be detected in coincidence with high sensitivity, thus yielding a spatial resolution in the mm-range. Although a high number of radiotracers has been developed only a limited number are commonly used as a result of their sometimes cumbersome and difficult synthesis.

The short half-life of many PET-nuclides (^18^F t_½_ = 109.7 min) makes it necessary to produce the radiotracer on site, resulting in high investment costs. The effort for a radiotracer synthesis is nearly independent of the number of patient doses produced per synthesis run. Therefore, PET-centers usually focus on the application of well-established radiotracers such as the glucose derivative ^18^F[FDG](^18^F-2-fluoro-2-desoxyglucose) and only a few large PET-centers are able to provide a large number of other tracers. To bridge this gap, the development of new labeling techniques for the easy introduction of fluorine-18 into radiotracers without costly equipment would be favorable. A promising approach to simplify the radionuclide introduction significantly is the exploitation of the strong silicon-fluorine (Si–F) bond [1,2]. The silicon fluoride acceptor (SiFA) method, based on the efficient isotopic non-radioactive ^19^F for radioactive ^18^F exchange at the silicon atom, was recently developed and applied for simple one- and two-step ^18^F-fluorinations of peptides [3]. The radiosynthesis of different SiFA-derivatized peptides (RGD-, octreotate-, as well as a bombesin-analogue) resulted in specific activities of up to 680 GBq/µmol (18.4 Ci/µmol) for the final radiotracers, surprisingly high for a carrier added radiosynthesis [4]. This finding can be explained by DFT (density functional theory) model calculations. The most convenient feature of this SiFA labeling technique is that a final HPLC purification of the radiotracer from the precursor is not necessary, since labeling precursor and labeled product are identical. Another approach with Si-^18^F bearing building blocks was used for the *in vivo* evaluation of a Si-^18^F-derivatized bombesin derivative in tumor-bearing rodents [5,6]. However, a heating—as well as an HPLC-purification step—were necessary to obtain the final ^18^F-labeled compound in high specific activities. Most recently, the implementation of new functionalized SiFAs for the kit-like ^18^F-labeling of biomolecules was reported [7,8,9,10]. In *in vivo* studies, the use of SiFA-amounts as low as a few nanomoles resulted in ^18^F-labeled proteins with specific activities of up to 10–50 GBq/µmol (270–1,350 Ci/mmol), which would be suitable for receptor-imaging with PET. In this particular study, we aimed at evaluating the applicability of the SiFA technique for the derivatization of small molecule radiotracers, such as the D_2_ receptor ligands fallypride (FP, **1**), desmethoxyfallypride (DMFP, **2**) and raclopride (**3**). It was expected that the original SiFA building block, which cannot be extensively modified without losing its stability against hydrolysis, might have a detrimental influence on the binding affinity of the SiFA derivatized D_2_ receptor ligands. Several new Si-F bearing derivatives derived from basic model compounds were analyzed recently and evaluated as to their stability in aqueous solution with regard to the substitution pattern at the silicon atom [11]. The results are consistent with our previous findings that at least two sterically hindered substituents at the silicon atom are necessary to preserve the stability of the silicon-fluorine bond *in vitro* [3].

**Figure 1:**
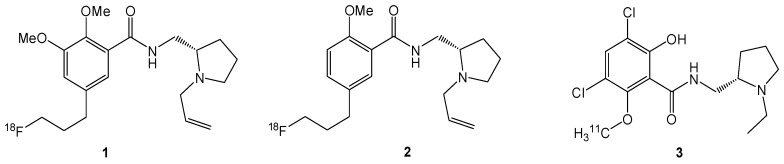
D_2_-receptor affine benzamide-derivatives used for PET-neuroimaging: [^18^F]-Fallypride (FP, **1**); [^18^F]-Desmethoxyfallypride (DMFP, **2**); [^11^C]-Raclopride (**3**).

With respect to these steric requirements, SiFA derived model compounds of commonly used PET imaging agents were synthesized to evaluate the potential of the SiFA-concept for the syntheses of SiFA-type small molecule radiotracers. The benzamide derivatives [^18^F]fallypride (**1**), [^18^F]-desmethoxyfallypride (**2**) and [^11^C]raclopride (**3**) radiotracers used for the PET-imaging of the dopaminergic system, were chosen as model compounds for this study [12,13] (Figure 1). All compounds are D_2_-receptor antagonists, which differ mainly in the receptor affinity, in the nanomolar (desmethoxyfallypride, raclopride) and picomolar range (fallypride), respectively [14]. These imaging agents are used for the diagnosis of different neurological disorders related to the dopaminergic system such as parkinsonism and craving [15,16].

**Figure 2:**
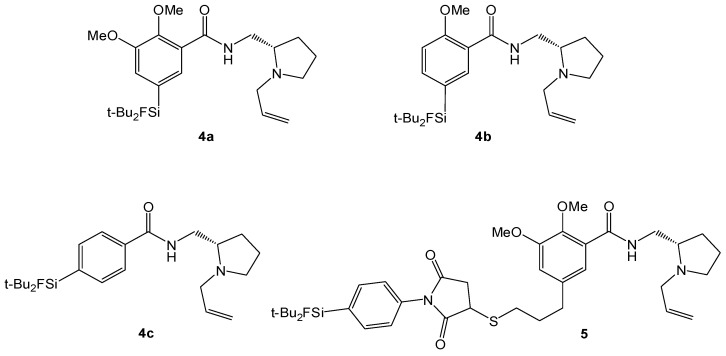
Synthesized SiFA-conjugated Fallypride-derivatives **4a–c**, **5**.

Due to the bulkiness of the SiFA-moiety that has to be introduced into the potential radiotracer, the ligand fallypride, having one of the highest affinities to the D_2_ receptor, was tested first as a scaffold for SiFA derivatization since even a certain loss of target affinity would not necessarily result in an unusable PET radiotracer. The two different strategies applied were: (i) the integration of the SiFA building block into the fallypride/desmethoxyfallypride general structure and (ii) the coupling of an already existing SiFA compound, namely SiFA-maleimide (SiFA-M, [17]), to a fallypride derivatized with an SH moiety at the butyl side chain (Figure 2). Besides the prerequisite of a good binding affinity to the targeted D_2_-receptor, the radiolabeling has to yield a radiotracer with a sufficiently high specific activity for D_2_ receptor imaging with PET. In order to simplify the radiosynthesis of the desired ^18^F fluorinated radiotracer, we only studied one-step radiosyntheses using the non-radioactive standards directly as the labeling precursors (isotopic exchange reaction). Hence, the amount of the precursor used determines the specific activity (in relation to the amount of radioactivity used for labeling).

The use of 10 nanomoles precursor would result in specific activities comparable to those of conventionally synthesized ^18^F radiotracers. If, e.g., 500 MBq (13.5 mCi) ^18^F are incorporated into 10 nmol of the labeling precursor the resulting specific activity would be as high as 50 GBq/µmol (1,350 Ci/mmol). Consequently, the identification of the lowest limit of the ratio precursor amount/concentration necessary for a sufficient radiochemical yield (introduction of fluorine-18) was the second important focus of this work.

## 2. Results and Discussion

### 2.1. Precursor Syntheses

**Scheme 1:**
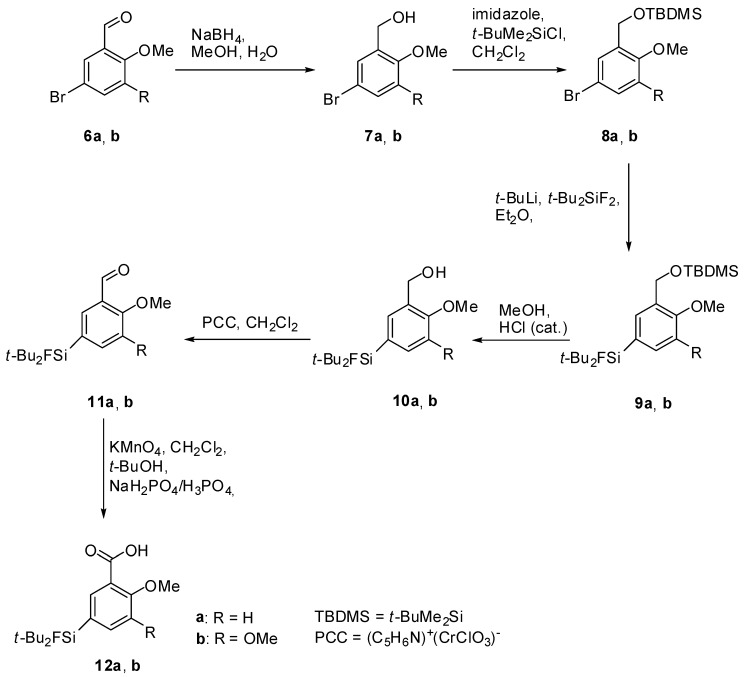
Synthesis of the silicon-modified carboxylic acids **12a** and **12b**.

**Figure 3:**
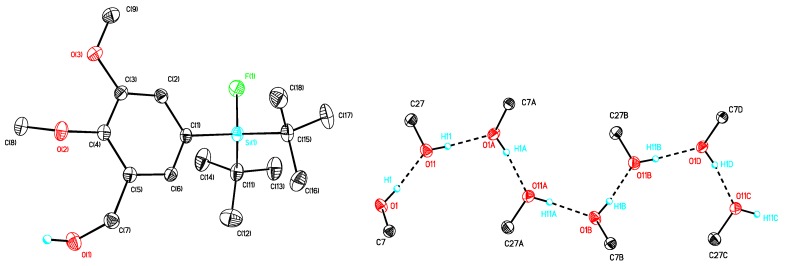
Molecular structure of **10b** (left) and simplified representation of the intermolecular hydrogen bridges (right). Atomic displacement parameters are drawn at 30% probability level.

**Figure 4:**
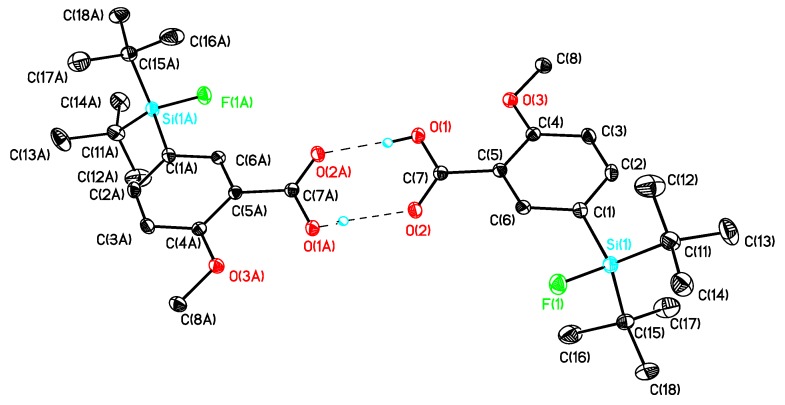
Molecular structure of **12a**. Atomic displacement parameters are drawn at 30% probability level.

**Figure 5:**
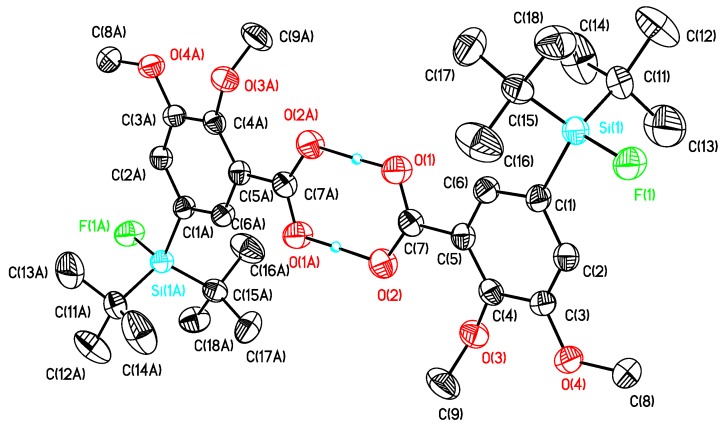
Molecular structure of **12b**. Atomic displacement parameters are drawn at 30% probability level.

The preparation of the SiFA-modified carboxylic acids **12a** and **12b** (Scheme 1) followed a general method described in our previous work [17]. Single crystals of compounds **10b**, **12a** and **12b** suitable for X-ray diffraction analysis were obtained as colorless needles by re-crystallization from diethyl ether/hexane. The molecular structures of these compounds are presented in Figure 3, Figure 4, Figure 5, and selected bond distances and bond angles are collected in Table 1.

All compounds crystallized monoclinically with eight (**10b**) or four (**12a**, **12b**) molecules in the unit cell. There are two crystallographically independent molecules in the unit cell of compound **10b** (Figure 3) with one of them being disordered. In Table 1, only the data for the non-disordered molecule are given. The silicon atoms in these compounds are four-coordinate and show each a distorted tetrahedral configuration with average angles of 109.59 (**10b**), 109.70 (**12a**) and 109.04 (**12b**). The largest deviations from the tetrahedral angle are found for C(11)–Si–C(15) (119.40°, **12b**) and F–Si–C(11) (104.15°, **12a**).

The Si-F distances are similar and fall in the range between 1.6033(11) (**12b**) and 1.6133(14) (**10b**) Å. They are slightly longer as compared to *t*-BuPh_2_SiF (1.6004(10) Å) but close to the Si–F distances in other SiFA-compounds [17]. Interestingly, intramolecular O(1)–HLO(11A) and O(11)–HLO(1) hydrogen bridges (Table 1) link the molecules of compound **10b** to form a one-dimensional polymer chain as presented in Figure 3. A noteworthy feature is the asymmetric intramolecular O(1)–HLO (2A) hydrogen bridge that links two molecules of **12a** and **12b**, respectively, to give a dimer (Table 2). Such hydrogen bridges are common for solid state structures of *p*-silylarylcarbonic acids [17].

**Table 1:** Selected bond lengths [Å] and angles [°] for **10b**, **12a**, **12b**.

Bond lengths [Å]	10b	12a	12b
Si(1)-F(1)	1.6133(13)	1.6125 (10)	1.6033 (11)
Si(1)-C(1)	1.8712(19)	1.8696 (15)	1.8695 (17)
Si(1)-C(11)	1.893(2)	1.8846 (16)	1.872 (2)
Si(1)-C(15)	1.888(2)	1.8911 (17)	1.875 (2)
**Bond angles [°]**			
F(1)-Si(1)-C(1)	104.78(8)	105.12 (6)	104.67 (8)
F(1)-Si(1)-C(11)	104.97(9)	104.15 (7)	105.61 (8)
F(1)-Si(1)-C(15)	105.23(9)	105.20 (6)	104.81 (8)
C(1)-Si(1)-C(11)	112.55(9)	112.36 (7)	109.04 (9)
C(1)-Si(1)-C(15)	109.59(9)	109.70 (7)	111.99 (9)
C(11)-Si(1)-C(15)	118.44(10)	118.96 (7)	119.40 (10)

**Table 2:** Hydrogen-bonding geometry [Å, °].

Compound	D-H...A	d(D-H)	d(H...A)	d(D...A)	<(DHA)
**10b**	O(1)-H(1)...O(11A)	0.82(3)	1.87(3)	2.682(2)	168(2)
**10b**	O(11)-H(11)...O(1)	0.83(3)	1.86(3)	2.695(2)	178(3)
**12a**	O(1)-H(1)...O(2A)	0.80(2)	1.84(2)	2.641(2)	173(2)
**12b**	O(1)-H(1)...O(2A)	1.22(4)	1.42(4)	2.640(2)	177(3)

**Scheme 2:**
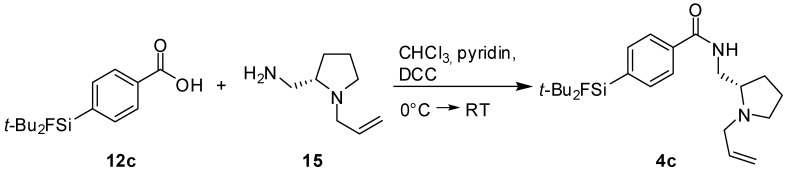
Coupling reaction of the SiFA-carboxylic acid **12c** to the (*S*)-(1-allyl-pyrrolidin-2-yl)methyl-amine.

**Scheme 3:**
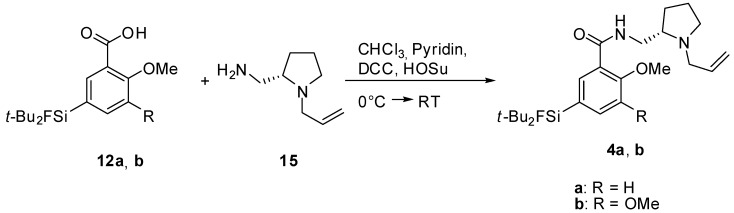
Coupling reaction of the SiFA-carboxylic acids to the (*S*)-(1-allyl-pyrrolidin-2-yl)methyl-amine in the presence of *N*-hydoxy-succinimide.

The test reaction for the coupling of carboxylic acids to (*S*)-(1-allylpyrrolidin-2-yl)methanamine was performed according to a literature procedure [12], using the carboxylic acid **12c** [17] (Scheme 2). Dicyclohexylcarbodimide (DCC) was used for the activation of the carboxylic acid and the fallypride-derivative was obtained in poor yield of 11%. In order to achieve a higher yield, the more powerful activating agent *N*-hydroxysuccinimide (HO-Su) was added to the reaction mixture (Scheme 3). SiFA-DMFP **4a** and SiFA-FP **4b** were obtained in yields of up to 41% after purification via column chromatography. Both compounds were characterized via two-dimensional NMR spectroscopy and high resolution mass spectrometry. In the latter, the molecular peak was found with high precision.

SiFA-M (1-(4-(di-tert-butylfluorosilyl)phenyl)-1H-pyrrole-2,5-dione) was synthesized according to a recently described procedure [17] and reacted with FP-Thiol [(*S*)-*N*-((1-allylpyrrolidin-2-yl)methyl)-5-(3-mercaptopropyl)-2,3-dimethoxybenzamide] in a water/acetonitrile mixture at pH 7.2. This Michael-addition was monitored via analytical HPLC and was completed within 10 min at ambient temperature. After semipreparative HPLC purification and lyophilization, the product SiFA-M-FP, **5**, was isolated in 49% yield and identified via ESI-MS and NMR spectroscopy.

### 2.2. *In Vitro* Evaluation

Compounds **4a–c** and **5** (SiFA-DMFP **4a**, SiFA-FP **4b**, SiFA-DDMFP **4c** and SiFA-M-FP **5**) were tested for their affinity towards the human D_2_ receptor (Table 3). All tested compounds showed *K*_i_-values in the nanomolar range. As expected for the derivatization of a small molecule with the bulky SiFA-moiety, the affinities are reduced by factors ranging between 22 [SiFA-DMFP **4a**compared to desmethoxyfallypride (**2**)], 44 [SiFA-M-FP **5** compared to fallypride (**1**)] and 342 [SiFA-FP **4b** compared to fallypride (**1**)]. Interestingly, SiFA-DMFP (**4a**) displays a higher affinity to the receptor compared to SiFA-FP (**4b**) and SiFA-DDMFP (**4c**). Among the newly developed substances **4a–c** and **5**, SiFA-M-FP (**5**) displays the highest binding affinity which might be explained with the higher distance of the derivatization site (SiFA is bound to the butyl side chain) to the receptor binding parts of the molecule. The methoxy moiety, which distinguishes the high affinity tracer fallypride (**1**) from the medium-affinity tracer desmethoxyfallypride (**2**) leads to a loss of binding affinity in the pair SiFA-DMFP **4a**/SiFA-FP **4b** inversely to the situation with fallypride (**1**)/desmethoxyfallypride (**2**). We can therefore assume that the benzoic acid ring system still influences the binding affinity but binds most probably in a different conformation to the binding site at the D_2_-receptor. However, we chose fallypride (**1**) as a scaffold for this study, because even an unavoidable decrease in binding-affinity towards the D_2_-receptor might still lead to a medium affinity ligand comparable to raclopride (**3**—the gold standard for D_2_-receptor imaging with PET). Therefore, the direct comparison of SiFA-M-FP (**5**) to [^11^C]raclopride **[^11^C]-3**, which is most frequently used in clinical studies of the dopaminergic system, looked more encouraging: When comparing the medium-affinity D_2_-receptor radiotracer raclopride (*K*_i_ = 1.21 nM) to SiFA-M-FP (**5**; *K*_i_ = 4.21) and SiFA-DMFP (**4a**; *K*_i_ = 13.6 nM) the affinity is only reduced by the factor 3.5 and 11, respectively. Thus, a successful *in vivo* D_2_ receptor imaging using radiolabeled SiFA-M-FP (**5**) or SiFA-DMFP (**4a**) might be possible.

**Table 3:** D_2_-receptor affinities of the developed substances **4a–c**, **5**.

Compound	*K*_i_ (nM ± SEM *)
Fallypride	0.0965 ± 0.0153
Desmethoxyfallypride	0.630 ± 0.089
Raclopride	1.21 ± 0.43
SiFA-DMFP, **4a**	13.6 ± 4.3
SiFA-FP, **4b**	33.0 ± 7.6
SiFA-DDMFP, **4c**	62.7 ± 16.9
SiFA-M-FP, **5**	4.21 ± 0.41

* SEM: standard error of the mean.

### 2.3. Radiolabeling

Radiolabeling reactions based on isotopic exchange usually have the drawback of a limited specific activity (SA), as the precursor cannot be separated from the final product. It is therefore crucial for *in vivo* applications of the synthesized radiotracer to reduce the precursor amount to the absolute minimum. Our previous studies showed that for radiofluorinations of small molecules such as a SiFA-aldehyde, amounts of 1–5 nmol were sufficient to achieve high radiolabeling yields [4]. For the direct labeling of SiFA-derivatized peptides, precursor amounts of 10–25 nmol were necessary [18]. The highest radioactivity incorporation rates were observed when the SiFA radiolabeling was carried out in polar aprotic solvents such as acetonitrile or DMSO. As the SiFA-moiety gets hydrolyzed very quickly under basic conditions, we tested the following two mild labeling procedures in acetonitrile, DMF or DMSO: (a) Kryptofix2.2.2/potassium oxalate/^18^F^−^ and (b) tetrabutylammonium bicarbonate/^18^F^−^. Table 4 summarizes the labeling of SiFA-DMFP (**4a**) under different labeling conditions.

To keep the labeling procedure as simple and convenient as possible, we performed the purification step by using a reversed phase cartridge (SepPak C-18 light) for all tested combinations. We determined the ^18^F-incroporation of ^18^F^−^ into SiFA-DMFP (**4a**) after a 5 min reaction time at room temperature with analytical radio-HPLC using samples of the crude reaction mixture. After dilution with 10 mL 1M HEPES buffer at pH = 4.0 (used to prevent hydrolysis) the desired radiotracer was purified using a reversed-phased SepPak-cartridge. The SepPak purification was necessary to remove unreacted ^18^F^−^, solvent and K2.2.2/potassium oxalate or TBAHCO_3_ from the product. After washing the cartridge with water, the products could be eluted very efficiently with 1 mL ethanol and were subsequently diluted with isotonic saline. The radiochemical yields (RCY) were calculated to the start of the synthesis and the radiochemical purity (RCP) analyzed by analytical radio-HPLC. Using SiFA-DMFP (**4a**) as the precursor, we found radioactivity incorporations in acetonitrile using TBAHCO_3_ of up to 61% in the crude reaction mixture (Table 4) and an overall RCY after purification of up to 42%. Under these conditions, the specific activity was calculated to be in the range of 88 GBq/µmol (2.4 Ci/µmol) using a precursor amount of 5 nmol and at least 41 GBq/µmol (1.1 Ci/µmol) using 10 nmol precursor when starting from ~1GBq (27 mCi) ^18^F-fluoride. This is in the suitable range for D_2_-neuroreceptor imaging with PET. The radiochemical purity of all products was >96% after purification. Using DMF or DMSO, the ^18^F-radioactivity incorporation was drastically reduced to less than 25%. More importantly, the desired product could not be purified via cartridge separation. Likewise, the labeling procedure using K2.2.2 resulted in a lower relative ^18^F-incorporation as well as a lower RCY compared to the procedure using TBAHCO_3_.

**Table 4:** Radiolabeling of 5–10 nmol SiFA-DMFP (**4a**), 5 min at ambient temperature.

Labeling method (solvent)	Amount of precursor	^18^F-incorporation ± SD *	RCY ± SD *	RCP **
(a) K2.2.2 *** (acetonitrile)	10 nmol	25.3 ± 2.5%	16.7 ± 1.4%	>96%
(b) TBAHCO_3 _(acetonitrile)	10 nmol	60.7 ± 3.1%	41.8 ± 5.7%	>96%
(b) TBAHCO_3 _(acetonitrile)	5 nmol	57.2 ± 6.2%	34.6 ± 5.6%	>96%
(b) TBAHCO_3 _(DMSO)	10 nmol	10.2 ± 0.3%	n.d.	<60%
(b) TBAHCO_3 _(DMF)	10 nmol	21.4 ± 4.5%	n.d.	<75%

* SD: standard deviation, all experiments were independently performed at least three times; ** after cartridge purification. *** K2.2.2 = Kryptofix2.2.2.

Using these labeling conditions optimized for [^18^F]-SiFA-DMFP **[^18^F]-4a**, we found comparable radiolabeling yields and purities for [^18^F]-SiFA-FP (**[^18^F]-4b)** and [^18^F]-SiFA-DDMFP (**[^18^F]-4c)**, whereas the radiolabeling yields for SiFA-M-FP (**[^18^F]-5)** were significantly lower (Table 5).

**Table 5:** Radiolabeling of **4a–c** and **5**, 10 nmol, labeling method (b) in acetonitrile.

Compound	^18^F-incorporation ± SD *	RCY ± SD *	RCP
SiFA-DMFP, **4a**	60.7 ± 3.1%	41.8 ± 5.7%	>96%
SiFA-FP, **4b**	60.8 ± 2.5 %	47.3 ± 5.9%	>94%
SiFA-DDMFP, **4c**	54.2 ± 6.0%	48.4 ± 0.7%	>97%
SiFA-M-FP, **5**	16.6 ± 10.2%	n.d.	<50%

* SD: standard deviation, all experiments were independently performed at least three times.

Moreover, radiolabeled [^18^F]-SiFA-M-FP **[^18^F]-5** could not be purified via the SepPak separation method described above. We can only speculate that the chemical design of the phenolic ring system of SiFA-maleimide leads to a significantly different stability of the SiFA-moiety compared to the benzamides **4a–c**. This finding is in line with our previous observation that protein labeling using [^18^F]-SiFA-thiol bound to a maleimide-derivatized protein resulted in higher radiolabeling yields than a vice versa labeling of a thiol-derivatized protein with [^18^F]-SiFA-maleimide.

The chemical purity was determined for all products by analytical HPLC at 214 nm and showed no side products for all radiolabeled derivative swith a RCP > 94%. The chemical stability of [^18^F]-SiFA-DMFP ([^18^F]-**4a**), SiFA-FP ([^18^F]**-4b**) and SiFA-DDMFP ([^18^F]-**4c**) was determined over 4 h at room temperature by analytical radio-HPLC. Within this time span, no decomposition was detected. The plasma stability was determined for SiFA-DMFP ([^18^F]**4a**) over 90min displaying no degradation of the product.

## 3. Experimental

### 3.1. General

All solvents used for the syntheses of **4a–c** (SiFA-DMFP, SiFA-FP, SIFA-DDMFP) were purified by distillation from appropriate drying agents under argon atmosphere. Solvents and chemicals used in the synthesis of **5**, SiFA-M-FP, were of analytical grade and used without further purification. Chemicals and solvents used for the labeling experiments were purchased in the highest available grade and were used without further purification. The NMR experiments were carried out with Jeol AS500, Bruker DRX 400, Bruker DRX 300, and Varian Mercury 200 spectrometers. Chemical shifts (δ) are given in ppm and are referenced to the solvent peaks, with the usual values calibrated against tetramethylsilane (^1^H, ^13^C, ^29^Si) and CFCl_3_ (^19^F). High-resolution mass spectra were obtained by using a Finnigan MAT95Q mass spectrometer and a LTQ Orbitrap mass spectrometer (Thermo Electron) using acetonitrile as the mobile phase. FT infrared spectra were recorded using a Bruker IFS 28 spectrometer. Elemental analyses were performed on a LECO CHNS-932 analyzer. The analytical and semi-preparative HPLC system used was an Agilent 1200 system equipped with a raytest Gabi Star radioactivity detector together with a Chromolith Performance (RP-18e, 100–4.6 mm, Merck, Germany) and a Chromolith (RP-18e, 100–10 mm, Merck, Germany) column, respectively. SiFA-M was synthesized according to a published procedure [17]. The synthesis of the thiol-substituted fallypride will be described elsewhere.

### 3.2. Crystallography

Crystals of compounds **10b**, **12a** and **12b** suitable for single-crystal X-ray diffraction analyses were grown by re-crystallization from diethyl ether/*n*-hexane. Crystallographic data are summarized in Table 1. Intensity data were collected with a Nonius KappaCCD diffractometer with graphite-monochromated Mo-Kα radiation. The data collections covered almost the whole sphere of the reciprocal space with 3 (5), 4 (7 and 8b) sets at different κ angles and 227 (5), 339 (7), 494 (8b) frames by ω-rotation (Δ/ω = 1°) at 2 × 160 s (5), 80 s (7), 60 s (8b) per frame. Crystal decay was monitored by repeating the initial frames at the end of the data collection. After analysis of the duplicate reflections, there was no indication of any decay. The structures were solved by direct methods (SHELXS97 [19]). Refinement applied full-matrix least-squares methods (SHELXL97). All H atoms were located in the difference Fourier map and their positions were isotropically refined with Uiso constrained at 1.2 times Ueq of the carrier C atom for non-methyl and 1.5 times Ueq of the carrier C atom for methyl groups. Atomic scattering factors for neutral atoms and real and imaginary dispersion terms were taken from International Tables for X-ray Crystallography [20]. The figures were created by SHELXTL. Crystallographic data are given in Table 6. Crystallographic data for the structures reported in this paper have been deposited with the Cambridge Crystallographic Data Centre as commentary material publication No. CCDC-704771, CCDC-704583 and CCDC-704772. Copies of the data can be obtained free of charge on application to CCDC, 12 Union Road, Cambridge, CB2 1EZ, UK (Fax: +44 1223 336033; Email: deposit@ccdc.cam.ac.uk ).

**Table 6:** Crystallographic data for **10a**, **12a** and **12b**.

	10b	12a	12b
Empirical formula	C_17_H_29_FO_3_Si	C_16_H_25_FO_3_Si	C_17_H_27_FO_4_Si
Formula mass [g/mol]	328.49	312.45	342.48
Crystal system	monoclinic	monoclinic	monoclinic
Crystal size	0.44 × 0.28 × 0.10	0.40 × 0.28 × 0.20	0.28 × 0.24 × 0.20
Space group	P21/c	P21/n	P21/c
a [Å]	8.0498(3)	6.91101(14)	12.866(3)
b [Å]	35.1976(15)	16.975(3)	8.1150(16)
c [Å]	13.5997(7)	14.529(3)	19.412(4)
β [°]	102.644(2)	94.46(3)	108.51(3)
V [Å^3^]	3759.8(3)	1699.3(6)	1921.9(7)
Z	8	4	4
ρ_calcd_.[mg/m^3^]	1.161	1.221	1.184
μ [mm^−1^]	0.143	0.155	0.146
F(000)	1424	672	736
θ range [°]	1.64–27.79	2.78–27.48	2.74–27.48
Index ranges	−16 ≤ h ≤ 17	−8 ≤ h ≤ 8	−15 ≤ h ≤ 15
	−44 ≤ k ≤ 46	−22 ≤ k ≤ 22	−10 ≤ k ≤ 10
	−8 ≤ l ≤ 10	−18 ≤ l ≤ 18	−25 ≤ l ≤ 25
No. of reflections collected	26113	17951	17285
Completeness of θ_max_[%]	97.3	99.8	99.8
No. of independent reflections / R_int._	8677/0.037	3872/0.022	4401/0.022
No. of reflections observed with [I > 2σ(I)]	6140	2389	1602
No. of refined parameters	443	200	211
GoF(F^2^)	1.031	0.900	0.729
R_1_(F) [I > 2σ(I)]	0.0517	0.0362	0.0364
wR_2_(F^2^) (all data)	0.1409	0.0924	0.0905
(Δ/σ)_max_	0.001	0.000	0.000
Largest differnce peak/hole [e / Å^3^]	0.448/−0.251	0.199/−0.237	0.106/−0.171

### 3.3. Chemistry

(*5-Bromo-2-methoxyphenyl)methanol* (**7a**). A solution of sodium borohydride (0.94 g, 24.8 mmol, 1.0 equiv.) in H_2_O (30 mL) was added to a solution of the benzaldehyde derivative **6a** (5.33 g, 24.8 mmol) in methanol (200 mL). After stirring at room temperature for 24 h, methanol was evaporated and the aqueous residue was diluted with diethyl ether (200 mL). The aqueous phase was extracted with diethyl ether (3 × 100 mL), the combined organic layers were dried with MgSO_4_, filtered and the filtrate was evaporated to afford **7a** (5.06 g, 23.31 mmol, 94%) as colourless oil. ^1^H-NMR (400.13 MHz, CDCl_3_): δ (ppm) = 7.39 (d, ^4^*J*(1H-1H) = 2.4 Hz, 1H, C(6)H), 7.33 (dd, ^3^*J*(1H-1H) = 8.8 Hz, ^4^*J*(1H-1H) = 2.4 Hz, 1H, C(4)H), 6.72 (d, ^3^*J*(1H-1H) = 8.8 Hz, 1H, C(3)H), 4.61 (s, 2H, CH_2_), 3.81 (s, 3H, OCH_3_), 2.35 (s, 1H, OH).

*(5-Bromo-2,3-dimethoxyphenyl)methanol *(**7b**). The procedure was analogous to the synthesis of 7a. The benzaldehyde derivative **6b** (5.35 g, 21.83 mmol) gave alcohol **7b** (5.04 g, 20.40 mmol, 93%) as a colourless oil. ^1^H-NMR (200.13 MHz, CDCl_3_): δ (ppm) = 7.05 (d, ^4^*J*(1H-1H) = 2.3 Hz, 1H, C(4)H), 6.93 (d, ^4^*J*(1H-1H) = 2.3 Hz, 1H, C(6)H), 4.59 (d, ^3^*J*(1H-1H) = 5.6 Hz, 2H, CH_2_), 3.80 (s, 3H, OCH_3_), 3.78 (s, 3H, OCH_3_), 2.93 (t, ^3^*J*(1H-1H) = 5.6 Hz, 1H, OH).

*(5-Bromo-2-methoxybenzyloxy)(tert-butyl)dimethylsilane* (**8a**). To a solution of **7a** (5.00 g, 23.00 mmol) in dichloromethane (150 mL) imidazole (2.03 g, 29.89 mmol, 1.3 equiv.) was added and the suspension was stirred at ambient temperature for 10 min. *t*-Butyldimethylchlorosilane (*t*-BuMe_2_SiCl, 4.16 g, 27.59 mmol, 1.2 equiv.) was added and stirring was continued for 20 h. For work-up the mixture was diluted with H_2_O (40 mL) and the aqueous phase was extracted with dichloromethane (3 × 50 mL). The combined organic layers were dried with MgSO_4_, filtered and the solvent was evaporated to afford **8a** (7.10 g, 21.43 mmol, 93%) as a red oil. ^1^H-NMR (400.13 MHz, CDCl_3_): δ (ppm) = 7.57 (d, ^4^*J*(^1^H-^1^H) = 2.5 Hz, 1H, C(6)H), 7.29 (dd, ^4^*J*(^1^H-^1^H) = 2.5 Hz, ^3^*J*(^1^H-^1^H) = 8.6 Hz, 1H, C(4)H), 6.66 (d, ^3^*J*(^1^H-^1^H) = 8.6 Hz, 1H, C(3)H), 4.71 (s, 2H, CH_2_), 3.77 (s, 3H, OCH_3_), 0.97 (s, 9H, CCH_3_), 0.13 (s, 6H, SiCH_3_). ^13^C[1H]-NMR (100.63 MHz, CDCl_3_): δ (ppm) = 154.9 (s, C(2)), 132.2 (s, C(6)), 130.0 (s, C(4)), 129.5 (s, C(1)), 113.0 (s, C(3)), 111.1 (s, C(5)), 59.7 (s, CH2), 55.3 (s, OCH3), 26.0 (s, CCH3), 18.5 (s, C(CH3)3, −5.3 (s, SiCH3).

*(5-Bromo-2,3-dimethoxybenzyloxy)(tert-butyl)dimethylsilane* (**8b**). The procedure was analogous to the synthesis of 8a. The benzyl alcohol derivative **7b** (4.93, 19.95 mmol) gave the silylated alcohol **8b** (5.88g, 16.27 mmol, 82%) as a slightly yellowish oil. ^1^H-NMR (400.13 MHz, CDCl_3_): δ (ppm) = 7.08 (d, ^4^*J*(^1^H-^1^H) = 2.3 Hz, 1H, C(4)H), 6.82 (d, ^4^*J*(^1^H-^1^H) = 2.2 Hz, 1H, C(6)H), 4.62 (s, 2 H, CH_2_), 3.72 (s, 3H, OCH_3_), 3.67 (s, 3H, OCH_3_), 0.83 (s, 9H, CCH3), 0.00 (s, 6H, SiCH_3_).

*Di-tert-Butyl(3-((tert-butyldimethylsilyloxy)methyl)-4-methoxyphenyl)fluorosilane *(**9a**). To a stirred solution of **8a** (5.00 g, 15.09 mmol) in dry diethyl ether (150 mL), *t*-BuLi (17.8 mL, 1.7 mol/L, 30.18 mmol, 2.0 equiv.) was added dropwise at −78 °C. After stirring for 10 min *t*-Bu_2_SiF_2_ (3.00 g, 16.60 mmol, 1.1 equiv.) was added and stirring was continued for 19 h while the reaction mixture was allowed to warm to ambient temperature. The mixture was washed with H_2_O (50 mL) and the aqueous phase was extracted with diethylether (3 × 50 mL). The combined organic layers were dried with MgSO_4_, filtered and the solvent was evaporated to afford **9a** (5.77 g, 13.98 mmol, 93%) as a colourless oil. ^1^H-NMR (300.13 MHz, CDCl_3_): δ (ppm) = 7.64 (s, 1H, C(6)H), 7.38 (d, ^3^*J*(1H-1H) = 8.1 Hz, 1H, C(4)H), 6.73 (d, ^3^*J*(^1^H-^1^H) = 8.6 Hz, 1H, C(3)H), 4.69 (s, 2H, CH_2_), 3.70 (s, 3H, OCH_3_), 0.96 (s, 18H, CCH_3_), 0.85 (s, 9H, CCH_3_), 0.00 (s, 6H, SiCH_3_). ^13^C[1H]-NMR (75.48 MHz, CDCl_3_): δ (ppm) = 157.7 (s, C(2)), 134.4 (d, ^3^*J*(^13^C-^19^F) = 3.9 Hz, C(6)), 132.4 (d, ^3^*J*(^13^C-^19^F) = 4.5 Hz, C(4)), 129.3 (s, C(1)), 124.3 (d, ^2^*J*(^13^C-^19^F) = 13.9 Hz, C(5)), 109.2 (s, C(3)), 60.5 (s, CH_2_), 55.3 (s, OCH_3_), 27.8 (s, CCH_3_), 26.4 (s, CCH_3_), 20.8 (d, ^2^*J*(^13^C-^19^F) = 12.5 Hz, C(CH_3_)_3_), 18.8 (s, C(CH_3_)_3_, −4.9 (s, SiCH_3_). ^19^F-NMR (282.38 MHz, CDCl_3_): δ (ppm) = −189.1 (s, ^1^*J*(^19^F-^29^Si) = 297 Hz). ^29^Si-NMR (59.63 MHz, CDCl_3_): δ (ppm) = 20.6 (s, Si(CH_3_)_2_tBu), 14.7 (d, ^1^*J*(^29^Si-^19^F) = 297 Hz, SiFtBu_2_). Elemental analysis calculated (%) for C_22_H_41_FO_2_Si_2_ (412.73 g/mol): C 64.0, H 10.0; found (%): C 63.8, H 9.6. HR-MS (GC-EI): calculated for C_22_H_41_O_2_F^28^SNa^+^ 435.2521, found 435.2530 [M+Na^+^].

*Di-tert-Butyl(3-((tert-butyldimethylsilyloxy)methyl)-4,5-dimethoxyphenyl)-fluorosilane *(**9b**). To a stirred solution of **8b** (5.70 g, 15.77 mmol) in dry diethylether (150 mL) and dry THF (50 mL) *t*-BuLi (18.6 mL, 1.7 mol/L, 31.55 mmol, 2.0 equiv.) was added dropwise at −78 °C. After stirring for 5 min *t*-Bu_2_SiF_2_ (3.13 g, 17.35 mmol, 1.1 equiv.) was added and stirring was continued for 19 h while the reaction mixture was allowed to warm to ambient temperature. The mixture was concentrated in vacuo and the aqueous residue dissolved in chloroform. The aqueous phase was extracted with chloroform (4 × 100 mL) and the combined organic layers were concentrated in vacuo, dried with MgSO_4_ and filtered. The solvent was evaporated and the crude product was purified by column chromatography (hexane/diethylether = 20/1) to afford **9b** (4.74 g, 10.71 mmol, 68%) as a colorless oil. ^1^H-NMR (200.13 MHz, CDCl_3_): δ (ppm) = 7.33 (s, 1H, C(6)H), 7.07 (s, 1H, C(4)H), 4.82 (s, 2H, CH_2_), 3.89 (s, 3H, OCH_3_), 3.87 (s, 3H, OCH_3_), 1.08 (s, 18H, CCH_3_), 0.95 (s, 9H, CCH_3_), 0.11 (s, 6H, SiCH_3_). ^13^C[^1^H]-NMR (100.63 MHz, CDCl_3_): δ (ppm) = 151.5 (s, C(2)), 147.0 (s, C(3)), 134.4 (s, C(1)), 128.4 (d, ^2^*J*(^13^C-^19^F) = 13.7 Hz, C(5)), 125.1 (d, ^3^*J*(^13^C-^19^F) = 4.5 Hz, C(6)), 116.5 (d, ^3^*J*(^13^C-^19^F) = 3.9 Hz, C(4)), 60.5 (s, CH_2_ or OCH_3_), 60.1 (s, CH_2_ or OCH_3_), 55.8 (s, OCH_3_), 27.4 (s, CCH_3_), 25.9 (s, CCH_3_), 20.3 (d, ^2^*J*(^13^C-^19^F) = 12.4 Hz, C(CH_3_)_3_), 18.3 (s, C(CH_3_)_3_, −5.3 (s, SiCH_3_). ^19^F-NMR (282.38 MHz, CDCl_3_): δ (ppm) = −189.1 (s, ^1^*J*(^19^F-^29^Si) = 298 Hz). ^29^Si-NMR (59.63 MHz, CDCl_3_): δ (ppm) = 20.7 (s, Si(CH_3_)_2_*t*Bu), 14.3 (d, ^1^*J*(^29^Si-^19^F) = 298 Hz, SiF*t*Bu_2_). Elemental analysis calculated (%) for C_23_H_43_FO_3_Si_2_ (412.73 g/mol): C 62.4, H 9.8; found (%): C 62.2, H 9.6. HR-MS (GC-EI): calculated for C_23_H_43_O_3_F^28^SiNa^+^ 465.2627, found 465.2638 [M+Na^+^].

*(5-(di-tert-Butylfluorosilyl)-2-methoxyphenyl)methanol* (**10a**). To a stirred solution of **9a** (4.90 g, 11.88 mmol) in methanol (250 mL) catalytic amounts of concentrated HCl was added. After stirring at room temperature for 19 h, methanol was evaporated and the residue dissolved in H_2_O and diethyl ether. The aqueous phase was extracted with diethyl ether (3 × 50 mL) and the combined organic layers were washed with saturated NaHCO_3_-solution (50 mL), dried with MgSO_4_, and filtrated. The solvent was evaporated to afford **10a** (3.05 g, 10.22 mmol, 86%) as a colorless oil. ^1^H-NMR (400.13 MHz, CDCl_3_): δ (ppm) = 7.50 (d, ^3^*J*(1H-1H) = 8.1 Hz, 1H, C(4)H), 7.47 (s, 1H, C(6)H), 6.89 (d, ^3^*J*(1H-1H) = 8.1 Hz, 1H, C(3)H), 4.67 (d, ^3^*J*(1H-1H) = 6.2 Hz, 2H, CH_2_), 3.85 (s, 3H, OCH_3_), 2.51 (t, ^3^*J*(1H-1H) = 6.2 Hz, 1H, OH), 1.03 (s, 18H, CCH_3_). ^13^C[^1^H]-NMR (400.13 MHz, CDCl_3_): δ (ppm) = 158.7 (s, C(2)), 135.2 (d, ^3^*J*(^13^C-^19^F) = 4.3 Hz, C(6)), 134.3 (d, ^3^*J*(^13^C-^19^F) = 4.1 Hz, C(4)), 128.3 (s, C(1)), 124.5 (d, ^2^*J*(^13^C-^19^F) = 13.9 Hz, C(5)), 109.6 (s, C(3)), 62.2 (s, CH_2_), 55.0 (s, OCH_3_), 27.3 (s, CCH_3_), 20.2 (d, ^2^*J*(^13^C-^19^F) = 12.4 Hz, C(CH_3_)_3_). ^19^F-NMR (282.38 MHz, CDCl_3_): δ (ppm) = −189.0 (s, ^1^*J*(^19^F-^29^Si) = 297 Hz). ^29^Si-NMR (59.63 MHz, CDCl_3_): δ (ppm) = 14.6 (d, ^1^*J*(^29^Si-^19^F) = 297 Hz). Elemental analysis calculated (%) for C_16_H_27_FO_2_Si (298.47 g/mol): C 64.4, H 9.1; found (%): C 64.1, H 9.2. IR (KBr): ν (cm^−1^) = 3326 (ν(OH)). HR-MS (GC-EI): calculated for C_16_H_27_O_2_F^28^Si^+^ 298.1759, found 298.1761 [M^+^].

*(5-(di-tert-Butylfluorosilyl)-2,3-dimethoxyphenyl)methanol* (**10b**). The procedure was analogous to the synthesis of **10a** starting from the protected alcohol **9b** (4.69, 10.59 mmol). The crude product was purified by column chromatography (hexane/diethylether = 3/1 → hexane/diethylether = 2/1 → hexane/diethylether = 1/1 → hexane/diethylether) to afford **10b** (3.13, 9.53 mmol, 90%) as a white crystalline solid of m.p. 80 °C. ^1^H-NMR (200.13 MHz, CDCl_3_): δ (ppm) = 7.14 (d, ^4^*J*(^1^H-^1^H) = 0.9 Hz, 1H, (C4)H), 7.03 (d, ^4^*J*(^1^H-^1^H) = 0.9 Hz, 1H, (C6)H), 4.61 (s, 2H, CH_2_), 3.81 (s, 3H, OCH_3_), 3.80 (s, 3H, OCH_3_), 3.17 (s, 1H, OH), 1.00 (s, 18H, CCH_3_). ^13^C[^1^H]-NMR (75.48 MHz, CDCl_3_): δ (ppm) = 152.1 (s, (C2)), 148.6 (s, (C3)), 134.5 (s, (C1)), 129.3 (d, ^2^*J*(^13^C-^19^F) = 13.6 Hz, (C5)), 126.9 (d, ^3^*J*(^13^C-^19^F) = 4.1 Hz, (C4)), 117.7 (d, ^3^*J*(^13^C-^19^F) = 3.9 Hz, (C6)), 61.3 (s, CH_2_), 61.1 (s, OCH_3_), 56.1 (s, OCH_3_), 27.8 (s, CCH_3_), 20.6 (d, ^2^*J*(^13^C-^19^F) = 12.4 Hz, C(CH_3_)_3_). ^19^F-NMR (282.38 MHz, CDCl_3_): δ (ppm) = −188.5 (s, ^1^*J*(^19^F-^29^Si) = 298 Hz). ^29^Si-NMR (59.63 MHz, CDCl_3_): δ (ppm) = 14.1 (d, ^1^*J*(^29^Si-^19^F) = 298 Hz). Elemental analysis calculated (%) for C_17_H_29_FO_3_Si (328.19 g/mol): C 62.2, H 8.9; found (%): C 62.0, H 8.9. IR (KBr): ν (cm^−1^) = 3294 (ν(OH)). HR-MS (GC-EI): calculated for C_17_H_29_O_3_F^28^Si^+^ 328.1865, found 328.1869 [M^+^].

*(3-(Bromomethyl)-4-methoxyphenyl)di-tert-butylfluorosilane* (**10c**). The procedure was analogous to the synthesis of **10a** starting from **9a** (2.80 g, 6.78 mmol) and concentrated HBr (50 mL). After crystallisation from diethyl ether/hexane, **10c** (1.80 g, 4.98 mmol, 73%) was obtained as white crystalline solid of m.p. 61 °C. ^1^H-NMR (300.13 MHz, CDCl_3_): δ (ppm) = 7.54–7.57 (m, 2 H, C(4)H und C(6)H), 6.93 (d, ^3^*J*(^1^H-^1^H) = 8.7 Hz, 1H, C(3)H), 4.61 (s, 2H, CH_2_), 3.94 (s, 3H, OCH_3_), 1.08 (s, 18H, CCH_3_). ^13^C[^1^H]-NMR (75.48 MHz, CDCl_3_): δ (ppm) = 159.1 (s, C(2)), 137.1 (d, ^3^*J*(^13^C-^19^F) = 4.1 Hz, C(4)), 136.6 (d, ^3^*J*(^13^C-^19^F) = 4.2 Hz, C(6)), 126.1 (s, C(1)), 125.2 (d, ^2^*J*(^13^C-^19^F) = 14.1 Hz, C(5)), 110.8 (s, C(3)), 55.9 (s, OCH_3_), 29.5 (s, CH_2_), 27.8 (s, CCH_3_), 20.7 (d, ^2^*J*(^13^C-^19^F) = 12.4 Hz, C(CH_3_)_3_). ^19^F-NMR (282.38 MHz, CDCl_3_): δ (ppm) = −189.0 (s, ^1^*J*(^19^F-^29^Si) = 297 Hz). ^29^Si-NMR (59.63 MHz, CDCl_3_): δ (ppm) = 14.4 (d, ^1^*J*(^29^Si-^19^F) = 297 Hz). Elemental analysis calculated (%) for C_16_H_26_BrFOSi (360.09 g/mol): C 53.2, H 7.3; found (%): C 53.3, H 6.9. HR-MS (LC-ESI): calculated for C_16_H_26_O^79^BrF^28^Si [M]^+^ (*m/z*) 360.0915, found 360.0922; for C_16_H_26_O^81^BrF^28^Si [M]^+^ (*m/z*) 362.0894, found 362.0907.

*3-(Chloromethyl)-4-methoxyphenyl)-di-tert-butylfluorosilane* (**10d**). The procedure was analogous to the synthesis of **10a** starting from **9a** (1.99 g, 4.82 mmol, 1.0 equiv.) and concentrated HCl (50 mL). Crystallisation from diethylether/hexane gave the product **10d** (1.16 g, 3.66 mmol, 76%) as white crystalline solid of m.p. 64 °C. ^1^H-NMR (300.13 MHz, CDCl_3_): δ (ppm) = 7.54–7.49 (m, 2H, C(4)H und C(6)H), 6.89 (d, ^3^*J*(^1^H-^1^H) = 7.9 Hz, 1H, C(3)H), 4.64 (s, 2H, CH_2_), 3.85 (s, 3H, OCH_3_), 1.03 (s, 18H, CCH_3_). ^13^C[^1^H]-NMR (100.63 MHz, CDCl_3_): δ (ppm) = 158.5 (s, C(2)), 136.2 (d, ^3^*J*(^13^C-^19^F) = 4.1 Hz, C(4)), 136.1 (d, ^3^*J*(^13^C-^19^F) = 4.2 Hz, C(6)), 125.2 (s, C(1)), 124.6 (d, ^2^*J*(^13^C-^19^F) = 14.1 Hz, C(5)), 110.1 (s, C(3)), 55.3 (s, OCH_3_), 41.6 (s, CH_2_), 27.3 (s, CCH_3_), 20.2 (d, ^2^*J*(^13^C-^19^F) = 12.5 Hz, C(CH_3_)_3_). Elemental analysis calculated (%) for C_16_H_26_ClFOSi (316.91 g/mol): C 60.6, H 8.3; found (%): C 60.8. HR-MS (LC-ESI): calculated for calculated for C_16_H_26_OF^28^Si [M−Cl^−^]^+^ (*m/z*) 281.1732, found 281.1733.

*5-(di-tert-Butylfluorosilyl)-2-methoxybenzaldehye *(**11a**). To an ice-cooled and stirred suspension of pyridinium chlorochromate (2.86 g, 13.27 mmol, 3.0 equiv.) in dry CH_2_Cl_2_ (150 mL) was added drop-wise within 10 min a solution of **10a** (1.32 g, 4.42 mmol) in dry CH_2_Cl_2_ (30 mL). The reaction mixture was stirred at room temperature for 2 h. After the reaction mixture had been diluted with diethyl ether (300 mL), the supernatant solution was decanted and the residue was washed with diethyl ether (2 × 100 mL). Filtration of the combined organic layers over a pad of silica and evaporation of the solvent afforded **11a** (1.31 g, 4.42 mmol, quantitative) as a slightly yellowish amorphous solid, m.p. 38 °C. ^1^H-NMR (400.13 MHz, CDCl_3_): δ (ppm) = 10.43 (s, 1H, CHO), 8.00 (s, 1H, C(6)H), 7.73 (d, ^3^*J*(^1^H-^1^H) = 8.3 Hz, 1H, C(4)H), 6.98 (d, ^3^*J*(^1^H-^1^H) = 8.3 Hz, 1H, C(3)H), 3.88 (s, 3H, OCH_3_), 0.98 (s, 18H, CCH_3_). ^13^C[^1^H]-NMR (100.63 MHz, CDCl_3_): δ (ppm) = 189.6 (s, CHO), 162.7 (s, C(2)), 141.6 (d, ^3^*J*(^13^C-^19^F) = 4.1 Hz, C(4)), 134.1 (d, ^3^*J*(^13^C-^19^F) = 4.3 Hz, C(6)), 129.9 (s, C(1)), 124.8 (d, ^2^*J*(^13^C-^19^F) = 14.1 Hz, C(5)), 111.1 (s, C(3)), 55.4 (s, OCH_3_), 27.2 (s, CCH_3_), 20.1 (d, ^2^*J*(^13^C-^19^F) = 12.4 Hz, C(CH_3_)_3_). ^19^F-NMR (282.38 MHz, CDCl_3_): δ (ppm) = −188.6 (s, ^1^*J*(^19^F-^29^Si) = 298 Hz). ^29^Si-NMR (59.63 MHz, CDCl_3_): δ (ppm) = 14.3 (d, ^1^*J*(^29^Si-^19^F) = 298 Hz). Elemental analysis calculated (%) for C_16_H_27_FO_2_Si (296.45 g/mol): C 64.8, H 8.5; found (%): C 64.0, H 8.7. HR-MS (GC-EI): calculated for C_16_H_25_O_2_F^28^Si [M]^+^ (*m/z*): 296.1602; found 296.1601.

*5-(di-tert-Butylfluorosilyl)-2,3-dimethoxybenzaldehyde* (**11b**). The procedure was analogous to the synthesis of 11a. The alcohol **10b** (3.04 g, 9.25 mmol) gave the aldehyde **11b** (3.12 g, 9.25 mmol, quantitative) as a slightly yellowish amorphous solid of m.p. 73 °C. ^1^H-NMR (400.13 MHz, CDCl_3_): δ (ppm) = 10.36 (s, 1H, CHO), 7.55 (s, 1H, C(6)H), 7.28 (s, 1H, C(4)H), 3.93 (s, 3H, OCH_3_), 3.84 (s, 3H, OCH_3_), 0.97 (s, 18H, CCH_3_). ^13^C[^1^H]-NMR (100.63 MHz, CDCl_3_): δ (ppm) = 189.9 (s, CHO), 153.6.7 (s, C(2)), 152.3 (s, C(3)), 129.2 (d, ^2^*J*(^13^C-^19^F) = 14.2 Hz, C(5)), 128.9 (s, C(1)), 124.7 (d, ^3^*J*(^13^C-^19^F) = 4.4 Hz, C(4)), 122.9 (d, ^3^*J*(^13^C-^19^F) = 4.2 Hz, C(6)), 62.0 (s, OCH_3_), 55.4 (s, OCH_3_), 27.1 (s, CCH_3_), 20.1 (d, ^2^*J*(^13^C-^19^F) = 12.2 Hz, C(CH_3_)_3_). ^19^F-NMR (282.38 MHz, CDCl_3_): δ (ppm) = −188.2 (s, ^1^*J*(^19^F-^29^Si) = 298.9 Hz). ^29^Si-NMR (59.63 MHz, CDCl_3_): δ (ppm) = 13.9 (d, ^1^*J*(^29^Si-^19^F) = 299.0 Hz). Elemental analysis calculated (%) for C_17_H_27_FO_3_Si (326.48 g/mol): C 62.5, H 8.3; found (%): C 62.4, H 8.4. IR (KBr): ν (cm-1) = 1692 (ν (C=O)). HR-MS (GC-EI): calculated for C_17_H_27_O_3_FSi [M]^+^ (*m/z*): 326.1708; found 326.1697.

*5-(di-tert-Butylfluorosilyl)-2-methoxybenzoic acid* (**12a**). To a stirred solution of **11a** (1.31 g, 4.42 mmol) in CH_2_Cl_2_ (5 mL) and *t*-BuOH (30 mL) one after another a buffered solution (30 mL, pH = 3) of NaH_2_PO_4_ (1.25 M), concentrated H_3_PO_4_ and an aqueous solution of KMnO_4_ (50 mL, 1 M) were added. Stirring was continued at room temperature for 3 h. The reaction mixture was quenched with saturated Na_2_SO_3_-solution (150 mL) and HCl (2 M). The latter reagents were added until the mixture turned colorless. After extraction with diethylether (3 × 200 mL) the combined organic layers were dried with MgSO_4_, filtered and the solvent was evaporated to afford **12a** (1.01 g, 3.23 mmol, 73%) as a white amorphous solid that was re-crystallised from diethyl ether/hexane to give colorless crystals of m.p. 123 °C. ^1^H-NMR (200 MHz, CDCl_3_): δ (ppm) = 8.40 (d, ^4^*J*(^1^H-^1^H) = 1.8 Hz, 1H, C(6)H), 7.81 (dd, ^3^*J*(1H-1H) = 8.3 Hz, ^4^*J*(^1^H-^1^H) = 1.8 Hz, 1H, C(4)H), 7.09 (d, ^3^*J*(^1^H-^1^H) = 8.3 Hz, 1H, C(3)H), 4.09 (s, 3H, OCH_3_), 1.04 (s, 18H, CCH_3_). ^13^C[^1^H]-NMR (100.63 MHz, CDCl_3_): δ (ppm) = 165.5 (s, COOH), 159.8 (s, C(2)), 140.8 (d, ^3^*J*(^13^C-^19^F) = 4.0 Hz, C(4)), 139.2 (d, ^3^*J*(^13^C-^19^F) = 4.4 Hz, C(6)), 127.1 (d, ^2^*J*(^13^C-^19^F) = 14.3 Hz, C(5)), 116.9 (s, C(1)), 111.1 (s, C(3)), 56.5 (s, OCH_3_), 27.2 (s, CCH_3_), 20.1 (d, ^2^*J*(^13^C-^19^F) = 12.2 Hz, C(CH_3_)_3_). ^19^F-NMR (282.38 MHz, CDCl_3_): δ (ppm) = −188.5 (s, ^1^*J*(^19^F-^29^Si) = 298 Hz). Elemental analysis calculated (%) for C_16_H_25_FO_3_Si (312.45 g/mol): C 61.5, H 8.1; found (%): C 61.1, H 7.9. HR-MS (GC-EI): calculated for C_16_H_25_O_3_FSi [M]_+_ (*m/z*): 312.1552; found 312.1563. IR (KBr): ν (cm^−1^) = 2989 (ν(OH)), 1701 (ν (C=O)).

*5-(di-tert-Butylfluorosilyl)-2,3-dimethoxybenzoic acid* (**12b**). The procedure was analogous to the synthesis of 12a. The aldehyde **11b** (2.00 g, 6.13 mmol) gave after re-crystallization from diethylether/hexane the carboxylic acid **12b** (2.02 g, 5.90 mmol, 96%) as colorless crystals of m.p. 118 °C. ^1^H-NMR (400.13 MHz, CDCl_3_): δ (ppm) = 7.91 (s, 1H, C(6)H), 7.34 (s, 1H, C(4)H), 4.10 (s, 3H, OCH_3_), 3.92 (s, 3H, OCH_3_), 1.04 (s, 18H, CCH_3_). 13C[1H]-NMR (100.63 MHz, C_6_D_6_): δ (ppm) = 166.8 (s, COOH), 152.4 (s, C(2)), 150.2 (s, C(3)), 130.1 (d, ^2^*J*(^13^C-^19^F) = 14.2 Hz, C(5)), 129.3 (d, ^3^*J*(^13^C-^19^F) = 4.7 Hz, C(4)), 123.3 (s, C(1)), 122.0 (d, ^3^*J*(^13^C-^19^F) = 3.9 Hz, C(6)), 61.1 (s, OCH_3_), 55.2 (s, OCH_3_), 27.1 (s, CCH_3_), 20.1 (d, ^2^J(^13^C-^19^F) = 12.1 Hz, C(CH_3_)_3_). ^19^F-NMR (282.38 MHz, CDCl_3_): δ (ppm) = −187.7 (s, ^1^*J*(^19^F-^29^Si) = 299 Hz). ^29^Si-NMR (59.63 MHz, CDCl_3_): δ (ppm) = 14.3 (d, ^1^*J*(^29^Si-^19^F) = 299 Hz). Elemental analysis calculated (%) for C_17_H_27_O_4_FSi (342.17 g/mol): C 59.6, H 8.0; found (%): C 59.4, H 7.6. HR-MS (GC-EI): calculated for C_17_H_27_O_4_FSi [M]^+^ (*m/z*): 342.1657; found 342.1641. IR (KBr): ν (cm^−1^) = 2974 (ν(OH)), 1685 (ν (C=O)).

*(S)-1-Allylpyrrolidine-2-carboxamide *(**14**). The synthesis followed the same procedure as described in the literature [12]. The reaction of (S)-pyrrolinamide **13** (1.03 g, 9.02 mmol) with allyl iodide (0.83 mL, 9.02 mmol, 1.0 equiv.) gave compound **14** (1.29 g, 8.37 mmol, 93%) as white amorphous solid. ^1^H-NMR (400.13 MHz, CDCl_3_): δ (ppm) = 7.19 (s, 1H, NH–H), 6.00 (s, 1H, NH–H), 5.87–5.76 (m, 1H, CH=CH_2_), 5.20–5.04 (m, 2H, CH=CH_2_), 3.28 (dd, ^3^*J*_1_(^1^H-^1^H) = 13.6 Hz, ^3^*J*_2_(^1^H-^1^H) = 5.9 Hz, 1H, CH), 3.14–3.07 (m, 1H, CH–H), 3.06–2.99 (m, 2H, N–CH_2_–CH), 2.37–2.27 (m, 1H, CH–H), 2.21–2.09 (m, 1H, CH–H), 1.91–1.81 (m, 1H, CH–H), 1.78–1.69 (m, 2H, CH_2_). ^13^C[^1^H]-NMR (100.63 MHz, CDCl_3_): δ (ppm) = 178.3 (s, CONH_2_), 135.1 (s, CH=CH_2_), 117.3 (s, CH=CH_2_), 66.7 (s, CH), 58.2 (s, CH_2_), 53.9 (s, CH_2_), 30.6 (s, CH_2_), 24.2 (s, CH_2_). HR-MS (LC-ESI): calculated for C_8_H_15_ON_2_ [M+H]^+^ (*m/z*): 155.1; found 155.2. 

*(S)-(1-Allylpyrrolidine-2-yl)methanamine* (**15**). The synthesis followed the same procedure as described in the literature [12]. The reaction of (*S*)-1-Allylpyrrolidine-2-carboxamide **14** (2.57 g, 16.67 mmol) with DIBAL-solution (100 mL, 1 M in THF, 6.0 eq.) gave compound **15** (1.68 g, 11.98 mmol, 72%) as slightly yellowish oil. ^1^H-NMR (400.13 MHz, CDCl_3_): δ (ppm) = 5.71–5.57 (m, 1H, CH=CH_2_), 4.98–4.80 (m, 2H, CH=CH_2_), 3.16 (dd, ^2^*J*_1_(^1^H-^1^H) = 13.4 Hz, ^3^*J*(^1^H-^1^H) = 5.5 Hz, 1H, NH_2_CH–H), 2.86–2.79 (m, 1H, CH), 2.60 (dd, ^2^*J*(^1^H-^1^H) = 13.5 Hz, ^3^*J*(^1^H-^1^H) = 7.5 Hz, 1H, NH_2_CH–H), 2.48–2.42 (m, 2H, CH_2_), 2.22–1.92 (m, 2H, NH_2_), 1.69–1.41 (m, 6H, CH_2_CH_2_CH_2_). ^13^C[^1^H]-NMR (100.63 MHz, CDCl_3_): δ (ppm) = 136.1 (s, CH=CH_2_), 116.2 (s, CH=CH_2_), 65.1 (s, CH), 57.5 (s, CH_2_), 54.1 (s, CH_2_), 44.2 (s, CH_2_NH_2_), 27.9 (s, CH_2_), 22.6 (s, CH_2_).

*(S)-N-((1-Allylpyrrolidine-2-yl)methyl)-5-(di-tert-butylfluorosilyl)-2-methoxybenzamide* (SiFA-DMFP **4a**). To an ice-cooled solution in dry CHCl_3_ containing the substituted benzoic acid **12a** (0.97 g, 3.10 mmol), (S)-(1-Allylpyrrolidine-2-yl)methanamine **15** (0.43 g, 3.10 mmol, 1.0 equiv.) and pyridine (0.25 mL, 3.10 mmol, 1.0 equiv.) dicyclohexylcarbodiimide (0.64 g, 3.10 mmol, 1.0 equiv.) and *N*-hydroxysuccinimide (0.36 g, 3.10 mmol, 1.0 equiv.) were added under stirring. The mixture was stirred at 0 °C for 5 h and 17 h at ambient temperature. After the white precipitate had been filtered, the filtrate was washed with saturated NaHCO_3_-solution (20 mL) and subsequently with H_2_O (20 mL). After extracting the aqueous phase with diethyl ether (20 mL) the combined organic layers were dried with MgSO_4_, filtered and the solvent was evaporated to give an oily residue. The latter was purified by column chromatography (CHCl_3_/Ethanol = 20/1 → CHCl_3_/Ethanol = 10/1) to afford benzamide **4a** (0.55 g, 1.27 mmol, 41%) as a yellowish oil. ^1^H-NMR (400.13 MHz, CDCl_3_): δ (ppm) = 8.44 (d, ^4^*J*(^1^H-^1^H) = 1.4 Hz, 1H, C(6)H), 8.37 (d, ^3^*J*(^1^H-^1^H) = 3.8 Hz, 1H, NH), 7.68 (dd, ^3^*J*(^1^H-^1^H) = 8.2 Hz, ^4^*J*(^1^H-^1^H) = 1.3 Hz, 1H, C(4)H), 7.00 (d, ^3^*J*(^1^H-^1^H) = 8.2 Hz, 1H, C(3)H), 5.96–5.84 (m, 1H, CH=CH_2_), 5.24-5.06 (m, 2H, CH=CH_2_), 3.95 (s, 3H, OCH_3_), 3.75 (ddd, ^2^*J*(^1^H-^1^H) = 13.8 Hz, ^3^*J*(^1^H-^1^H) = 7.1 Hz, ^3^*J*(^1^H-^1^H) = 3.1 Hz, 1H, NHCH–H), 3.48 (dd, ^2^*J*(^1^H-^1^H) = 13.5 Hz, ^3^*J*(^1^H-^1^H) = 5.3 Hz, 1H, CH_2_=CHCH-H), 3.35 (ddd, ^2^*J*(^1^H-^1^H) = 13.9 Hz, ^3^*J*(^1^H-^1^H) = 3.7 Hz, ^3^*J*(^1^H-^1^H) = 3.7 Hz, 1H, NHCH–H), 3.19–3.13 (m, 1H, NCH–H), 2.91 (dd, ^2^*J*(^1^H-^1^H) = 13.5 Hz, ^3^*J*(^1^H-^1^H) = 7.5 Hz, 1H, CH_2_=CHCH–H), 2.75 (s, 1H, CH), 2.33–2.22 (m, 1H, NCH–H), 1.99–1.85 (m, 1H, CH_2_CH_2_CH_2_), 1.79–1.61 (m, 3H, CHCH_2_CH–H), 1.02 (s, 18H, CCH_3_). ^13^C[^1^H]-NMR (100.63 MHz, CDCl_3_): δ (ppm) = 165.5 (s, CONH), 158.7 (s, C(2)), 138.6 (d, ^3^*J*(^13^C-^19^F) = 3.8 Hz, C(4)), 137.7 (d, ^3^*J*(^13^C-^19^F) = 4.7 Hz, C(6)), 135.8 (s, CH=CH_2_), 125.3 (d, ^2^*J*(^13^C-^19^F) = 14.5 Hz, C(5)), 120.9 (s, C(1)), 116.2 (s, CH=CH_2_), 110.7 (s, C(3)), 61.9 (s, CH), 57.0 (s, CH_2_CH=CH_2_), 55.5 (s, OCH_3_), 54.2 (s, NCH_2_), 41.3 (s, CH_2_NH), 28.5 (s, CH_2_), 27.3 (s, CCH_3_), 22.9 (s, CH_2_), 20.2 (d, ^2^*J*(^13^C-^19^F) = 12.0 Hz, C(CH_3_)_3_). ^19^F-NMR (282.38 MHz, CDCl_3_): δ (ppm) = −188.8 (s, ^1^*J*(^19^F-^29^Si) = 298 Hz). ^29^Si-NMR (59.63 MHz, CDCl_3_): δ (ppm) = 14.4 (d, ^1^*J*(^29^Si-^19^F) = 298 Hz). Elemental analysis calculated (%) for C_24_H_39_FN_2_O_2_Si∙H_2_O (452.68 g/mol): C 63.7, H 9.1, N 6.2; found: C 64.1, H 9.1, N 6.3. HR-MS (LC-ESI): calculated for C_24_H_40_O_2_N_2_FSi [M+H]^+^ (*m/z*): 435.2838; found: 435.2832.

*(S)-N-((1-Allylpyrrolidine-2-yl)methyl)-5-(di-tert-butylfluorosilyl)-2,3-dimethoxybenzamide* (SiFA-FP, **4b**). The procedure was analogous to the synthesis of 4a. The reaction of the benzoic acid derivative **12b** (1.47 g, 4.28 mmol) with (S)-(1-Allylpyrrolidine-2-yl)methanamine 15 (0.60 g, 3.10 mmol, 1.0 equiv.) gave SiFA-FP **4b** (0.81 g, 1.74 mmol, 41%) as a yellowish oil. ^1^H-NMR (400.13 MHz, CDCl_3_): δ (ppm) = 8.51 (s, br, 1H, NH), 7.93 (s, 1H, C(4)H), 7.24 (s, 1H, C(6)H), 5.99–5.87 (m, 1H, CH=CH_2_), 5.27-5.09 (m, 2H, CH=CH_2_), 3.93 (s, 3H, OCH_3_), 3.90 (s, 3H, OCH_3_), 3.80 (ddd, ^2^*J*(^1^H-^1^H) = 13.9 Hz, ^3^*J*(^1^H-^1^H) = 6.9 Hz, ^3^*J*(^1^H-^1^H) = 3.6 Hz, 1H, NHCH–H), 3.54 (dd, ^2^*J*(^1^H-^1^H) = 13.5 Hz, ^3^*J*(^1^H-^1^H) = 5.3 Hz, 1H, CH_2_=CHCH–H), 3.47–3.37 (m, 1H, NHCH–H), 3.19–3.13 (m, 1H, NCH–H), 2.98 (dd, ^2^*J*(^1^H-^1^H) = 13.2 Hz, ^3^*J*(^1^H-^1^H) = 7.6 Hz, 1H, CH_2_=CHCH–H), 2.86 (s, 1H, CH), 2.39–2.29 (m, 1H, NCH–H), 2.03–1.91 (m, 1H, CH_2_CH_2_CH_2_), 1.87–1.67 (m, 3H, CHCH_2_CH–H), 1.05 (s, 18H, CCH_3_). ^13^C[^1^H]-NMR (100.63 MHz, CDCl_3_): δ (ppm) = 165.5 (s, CONH), 151.9 (s, C(2)), 148.8 (s, C(3)), 134.6 (s, CH=CH_2_), 129.4 (d, ^2^*J*(^13^C-^19^F) = 14.0 Hz, C(5)), 128.4 (d, ^3^*J*(^13^C-^19^F) = 4.7 Hz, C(4)), 125.8 (s, C(1)), 120.3 (d, ^3^*J*(^13^C-^19^F) = 3.9 Hz, C(6)), 117.9 (s, CH=CH_2_), 62.2 (s, CH), 61.2 (s, OCH_3_), 57.0 (s, CH_2_CH=CH_2_), 56.0 (s, OCH_3_), 53.9 (s, NCH_2_), 41.0 (s, CH_2_NH), 28.4 (s, CH_2_), 27.3 (s, CCH_3_), 22.6 (s, CH_2_), 20.2 (d, ^2^*J*(^13^C-^19^F) = 12.2 Hz, C(CH_3_)_3_). ^19^F-NMR (282.38 MHz, CDCl_3_): δ (ppm) = −188.5 (s, ^1^*J*(^19^F-^29^Si) = 298 Hz). ^29^Si-NMR (59.63 MHz, CDCl_3_): δ (ppm) = 14.2 (d, ^1^*J*(^29^Si-^19^F) = 299 Hz). Elemental analysis calculated (%) for C_25_H_41_FN_2_O_3_Si∙H2O (482.70 g/mol): C 62.2, H 9.0, N 5.8; found (%): C 61.9, H 8.6, N 4.9. HR-MS (LC-ESI): calculated for C_25_H_42_O_3_N_2_FSi [M+H]^+^ (*m/z*): 465.2943; found: 465.2924.

*(S)-N-((1-Allylpyrrolidine-2-yl)methyl)-4-(di-tert-butylfluorosilyl) benzamide *(SiFA-DDMFP, **4c**). The procedure was analogous to the synthesis of **4a**. The reaction of the benzoic acid derivative **12c** [17] (0.50 g, 1.77 mmol) with (*S*)-(1-Allylpyrrolidine-2-yl)methanamine **15** (0.25 g, 1.77 mmol, 1.0 equiv.) gave SiFA-DDMFP **11c** (0.08 g, 0.20 mmol, 11%) as a yellowish oil. ^1^H-NMR (400.13 MHz, CDCl_3_): δ (ppm) = 7.80 (d, ^3^*J*(^1^H-^1^H) = 7.7 Hz, 2H, Ho), 7.69 (d, ^3^*J*(^1^H-^1^H) = 8.0 Hz, 2H, Hm), 7.00 (s, 1H, NH) 5.99–5.86 (m, 1H, CH=CH_2_), 5.29–5.12 (m, 2H, CH=CH_2_), 3.73 (ddd, ^2^*J*(^1^H-^1^H) = 13.7 Hz, ^3^*J*(^1^H-^1^H) = 7.4 Hz, ^3^*J*(^1^H-^1^H) = 3.2 Hz, 1H, NHCH–H), 3.50–3.43 (m, 2H), 3.2 (s, 1H), 3.04–2.76 (m, 2H), 2.42–2.28 (m, 1H), 1.92–1.90 (m, 1H), 1.64–1.54 (m, 3H), 1.06 (s, 18H, CCH_3_). ^13^C[^1^H]-NMR (100.63 MHz, CDCl_3_): δ (ppm) = 167.8 (s, CONH), 137.9 (d, ^2^*J*(^13^C-^19^F) = 13.7 Hz, Cp), 135.2 (s, Ci), 134.1 (d, ^3^*J*(^13^C-^19^F) = 4.7 Hz, Cm), 134.1 (s, CH=CH_2_), 126.0 (s, Co), 117.4 (s, CH=CH_2_), 62.8 (s, CH), 57.4 (s, CH_2_CH=CH_2_), 54.1 (s, NCH_2_), 40.7 (s, CH_2_NH), 28.2 (s, CH_2_), 27.2 (s, CCH_3_), 23.1 (s, CH_2_), 20.2 (d, ^2^*J*(^13^C-^19^F) = 12.3 Hz, C(CH_3_)_3_). ^19^F-NMR (282.38 MHz, CDCl_3_): δ (ppm) = −189.2 (s, ^1^*J*(^19^F-^29^Si) = 299 Hz). ^29^Si-NMR (59.63 MHz, CDCl_3_): δ (ppm) = 14.0 (d, ^1^*J*(^29^Si-^19^F) = 299 Hz). Elemental analysis calculated (%) for C_23_H_37_FN_2_OSi∙H_2_O + C_8_H_16_N_2_ (562.88 g/mol): C 66.2, H 9.5, N 8.4; found (%): C 66.0, H 9.2, N 8.2. HR-MS (LC-ESI): calculated for C_23_H_37_ON_2_FSi [M+H]^+^ (*m/z*): 405.2732; found: 405.2726.

*(S)-N-((1-Allylpyrrolidin-2-yl)methyl)-5-(3-(1-(4-(di-tert-butylfluorosilyl)phenyl)-2,5-dioxopyrrolidin-3-ylthio)propyl)-2,3-dimethoxybenzamide *(SiFA-M-FP, **5**). To a freshly prepared solution of 1-(4-(di-*tert*-butylfluorosilyl)phenyl)-1*H*-pyrrole-2,5-dione (SiFA-maleimide, [17]) (3 mg, 9 µmol) in phosphate buffer (PB, 0.1 M, pH 6.0) and acetonitrile (1:1) was added a solution of (*S*)-*N*-((1-allylpyrrolidin-2-yl)methyl)-5-(3-mercaptopropyl)-2,3-dimethoxybenzamide (FP-thiol) (3.4 mg, 9 µmol) in acetonitrile (100 µL) and the pH of the solution was adjusted to 7.2 using PB (0.1M, pH = 7.2, 200 µL). After 10 min, the product was isolated by semi-preparative HPLC applying a gradient of 40%–80% acetonitrile in 10 minutes. The product was obtained as white powder after lyophilization (3.1 mg, 4.4 µmol, 49% yield). ESI-MS calculated for [M+H]^+^ (*m/z*): 712.35, found: 712.36.

### 3.4. *In Vitro* Binding Assay

Evaluation of the dopamine D_2_-receptor binding affinity: The substances were dissolved in water or DMSO-water mixtures, respectively, and evaluated according to the [^3^H]spiperone binding protocol published by Malmberg and coworkers [21]. Source of the dopamine-D_2_-receptors was cell line HEK293 which expresses the D_2_ receptors stably. The *K*_d_-value of [^3^H]spiperone was determined in three saturation experiments (41.0 ± 6.0 pM (± SEM), p*K*_d_: 9.398 ± 0.067). All substances were evaluated in 3 independent competition experiments in six concentrations (each in triplicate). The *K*_i_-values were calculated from the IC_50_-values according to Cheng-Prusoff [22] as described in detail in [23].

### 3.5. Radiolabeling

*General procedure for the labeling of SiFA-compounds.* Aqueous [^18^F]fluoride (4000–7500 MBq) produced by the ^18^O(p,n)^18^F nuclear reaction on an enriched [^18^O]water (95%) target was loaded onto a Chromafix PS-HCO_3_ cartridge and eluted with a mixture of acetonitrile (800 µL), water (200 µL), potassium oxalate solution (1 M, 10 µL), and Kryptofix 2.2.2 (12.5 mg) (procedure a) or eluted with a mixture of 800 μL acetonitrile and 300 μL aqueous tetrabutylammonium hydrogen carbonate solution (0.075 mol/L, ABX, Radeberg, Germany) (procedure b). The solvents were removed by coevaporation to dryness under reduced pressure (650 mbar) using a stream of helium at 90 °C for 4 min. The drying step was repeated twice with acetonitrile (0.8 mL) (3 min) and full vacuum (~10 mbar) was applied in the final drying step (4 min). The dried [^18^F]F-complex was dissolved in dry acetonitrile, DMF or DMSO, respectively, (500–1000 µL) and used as stock solution for labeling. The labeling precursors (1–2 mg) were dissolved in dry acetonitrile, DMF or DMSO, respectively (1 µmol/mL) and aliquots containing 5–10 nmol of these stock solutions (5–10 µL) were used for labeling and incubated with 250 µL of the ^18^F stock solutions. After 5 min reaction time at ambient temperature (without stirring), samples were withdrawn from the reaction mixture and analyzed by analytical HPLC. For purification, the reaction mixture was diluted with 10 mL HEPES-buffer (1 M, pH = 4.0) and loaded on a SepPak C-18 light cartridge (Waters, Germany), preconditioned with 5mL ethanol and 5mL isotonic saline. The cartridge was washed with 10 mL isotonic saline and subsequently eluted with 1 mL ethanol. After dilution with 9 mL isotonic saline the radiotracers were measured, analyzed by radio-HPLC and used for plasma stability experiments.

*In vitro stability in human plasma.* To human plasma (500 µL) at 37 °C were added 10–100 MBq of the injectable solutions. The mixture was incubated at 37 °C. After 90 min, aliquots (75 µL, in triplicate) were removed and treated with acetonitrile (75 µL). Samples were then stored on ice for 5 min for complete precipitation of the plasma proteins. The precipitate was removed by centrifugation, and the supernatants were analyzed by radio-HPLC. Radioactivity in precipitate and supernatant was measured.

## 4. Conclusions

Motivated by our recent development of a kit-like radiolabeling strategy for the introduction of ^18^F-fluoride into proteins and target-specific peptides using Silicon-Fluoride-Acceptors (SiFAs) and their application in preclinical studies, we synthesized a series of SiFA-containing fallypride/ desmethoxyfallypride derivatives (Figure 2). Compared to other medium-affinity D_2_-receptor ligands such as DMFP **2**, the compounds showed a reduced affinity to the targeted receptor. However, the affinity is still in the nanomolar range and should therefore be high enough for an *in vivo* application. The radiochemical synthesis of the potential radiotracers, most notably the synthesis time of only 10 min and the easy cartridge purification, could be a breakthrough in radiofluorinations of small-molecule radiotracers. However, the potential of these SiFA-radiotracers remains to be shown in ongoing *in vitro* studies regarding receptor subtype-selectivity as well as Pgp-activity and finally in preclinical *in vivo* studies.
